# Associations of left ventricular systolic dysfunction with the factors among Thai patients on peritoneal dialysis: a cross-sectional study

**DOI:** 10.1186/s12882-019-1418-7

**Published:** 2019-07-12

**Authors:** Teeranan Angkananard, Jirayut Janma, Thanapath Wannasiri, Piyathida Sangthong, Siribha Changsirikulchai

**Affiliations:** 1 Division of Cardiovascular Medicine, Department of Medicine, Faculty of Medicine, HRH Princess Maha Chakri Sirindhorn Medical Center, Srinakharinwirot University, Nakhon Nayok, Thailand; 2 Division of Nephrology, Department of Medicine, Faculty of Medicine, HRH Princess Maha Chakri Sirindhorn Medical Center, Srinakharinwirot University, Nakhon Nayok, Thailand

**Keywords:** Peritoneal dialysis, Left ventricular systolic dysfunction, Neutrophil to lymphocyte ratio, Platelet to lymphocyte ratio, End stage renal disease

## Abstract

**Background:**

Factors associated with left ventricular systolic dysfunction (LVSD) of peritoneal dialysis (PD) patients are limited. We aim to explore and quantify the associated factors of LVSD among PD patients.

**Methods:**

Participants from a PD clinic treated between 2012 and 2014 at the HRH Princess Maha Chakri Sirindhorn Medical Center, Srinakharinwirot University, Nakhon Nayok, Thailand were recruited and divided into 2 groups according to their left ventricular ejection fraction (LVEF) (< 50% vs. ≥ 50%) with LVEF < 50% considered as LVSD. Correlations among the clinical, laboratory and echocardiographic variables were analyzed. The factors associated with LVSD were explored with univariate and multivariate logistic regression analyses. Beta coefficient along with odds ratio and 95% confidence interval (CI) were calculated and the *P* value < 0.05 was considered significant.

**Results:**

Among 103 subjects stratified as LVSD (*n* = 18, 17.5%). The mean (SD) age was 59.3 (12.7) years, and nearly halves were males. Preexisting CAD, diabetes (DM) and current smoking were 20 (19.4%), 63 (61.2%) and 23 (22.3%) patients, respectively. The median time of dialysis vintage was 12 (3, 24) months. Factors associated with LVSD and corresponding ORs with 95% CI by multivariate analysis were prior coronary artery disease (CAD) [5.08 (1.16, 22.19)], DM [6.36 (1.29, 31.49)], smoking [10.62 (2.17, 51.99)], neutrophil to lymphocyte ratio (NLR) > 3.6 [6.77 (1.41, 32.52)], and high serum phosphate [9.39 (2.16, 40.92)] were significantly associated with LVSD.

**Conclusions:**

Prior history of CAD, DM, smoking, high NLR and serum phosphate levels were found to be associated with LVSD for our PD patients. The evidence from prospective study is needed to confirm the predictive value of these variables.

**Electronic supplementary material:**

The online version of this article (10.1186/s12882-019-1418-7) contains supplementary material, which is available to authorized users.

## Background

Cardiovascular diseases (CVDs) are the leading causes of death in patients with end stage renal disease (ESRD) or on dialysis rather than progress to ESRD [1]. Atherosclerotic heart disease and congestive heart failure are the most common conditions and highest among patients performing dialysis [2, 3]. All causes of mortality increase six fold for ESRD patients with left ventricular systolic dysfunction (LVSD) [4]. The general population with preserved left ventricular systolic function was found to be a cardioprotective factor for cardiovascular morbidity [5]. Whereas, LVSD is a strong, unfavorable prognostic factor for hemodialysis (HD) and renal transplant patients [6–8]. The combination of established risk factors and left ventricular ejection fraction (LVEF) significantly increases the C index to anticipate both cardiovascular and all causes of mortality in HD patients [7]. The possible mechanisms are multifactorial. These include; preexisting ischemic heart disease, anemia, hyperparathyroidism, uremic toxins, an increased serum calcium-phosphate product, malnutrition and prolonged hemodynamic volume overload [9]. LVSD was observed in all dialysis patients at 5–36% which varies by the mode of dialysis and the echocardiography study time. The associated factors of LVSD in peritoneal dialysis (PD) patients are also limited [4, 7, 8, 10–14].

The inflammatory response is a key mechanism in the pathogenesis of atherosclerosis and its related clinical syndromes. Prior studies have proposed that inflammation may be responsible for LVSD and left ventricular diastolic dysfunction in PD patients [15, 16]. The neutrophil to lymphocyte ratio (NLR) and platelet to lymphocyte ratio (PLR), which are acquired from routine laboratory tests, have recently emerged as inflammatory biomarkers in ESRD patients [17, 18]. They have been found as useful predictors for LVSD in patients with acute coronary syndrome [19, 20]. Currently, there is no data of NLR and PLR for predicting LVSD in PD patients. The objectives of this study are those we have conducted a cross-sectional analysis to explore and quantify associated clinical factors and laboratory data, including NLR and PLR in conjunction with echocardiographic parameters, in PD patients with LVSD.

## Methods

### Study population

All PD patients treated between 2012 and 2014 from the PD clinic at the HRH Princess Maha Chakri Sirindhorn Medical Center, Srinakharinwirot University, Nakhon Nayok, Thailand were recruited. Our PD patients were prescribed only glucose-based PD solution. The most percentage of glucose-based PD solution was 1.5% dextrose. The 2.5 and 4.25% dextrose-based PD solution could be in a short term if patients had severe hypervolemia. The criteria for eligibility were patients at least 18 years of age without history of heart failure or pulmonary embolism, valvular heart disease, congenital heart disease, acute coronary syndrome, chronic lung and liver diseases, systemic lupus erythematosus, scleroderma, initiating PD treatment ≤2 weeks and infection during 3 months prior entry into this study. These data were verified by the physicians who were involved in our protocol. All patients have given their written, informed consent.

### Data collection

Baseline demographic and clinical data were collected. These include; age, gender, body mass index (BMI), body surface area (BSA), underlying diseases [CAD, hypertension, diabetes (DM), hyperlipidemia, and pulmonary disease], duration of PD therapy (dialysis vintage in months), current medications, history of smoking and alcohol use, systolic blood pressure and diastolic blood pressure levels. CAD is defined as history of receiving angioplasty, coronary artery bypass graft surgery, myocardial infarction or angina. BMI was calculated as weight/height^2^ (kg/m^2^).

Trans-thoracic echocardiography [21] was performed on the same day of enrollment by a single cardiologist (first author) and one of two sonographers (the third and fourth authors) using the Philip iE33 xMATRIX echocardiographic system and the criteria set forth by the American Society of Echocardiography [22, 23]. Volumetric measurement was calculated using two apical views: four- and two-chamber views, and manual tracing of the endocardial border at the end-diastole and end-systole of both views. End-diastole could be determined as the frame following mitral valve closure or at the onset of the QRS complex while end-systole was defined as the frame prior mitral valve opening. LVEF was then computed as follows:

$$ \mathrm{LVEF}=\left(\mathrm{LV}\ \mathrm{end}\ \mathrm{diastolic}\ \mathrm{volume}-\mathrm{LV}\ \mathrm{end}\ \mathrm{systolic}\ \mathrm{volume}|\mathrm{LV}\ \mathrm{end}\ \mathrm{diastolic}\ \mathrm{volume}\right) $$

By 2-D directed M-mode tracings, LV end-systolic diameter (LVESD) was estimated at end-systole, whereas LV posterior wall diastolic thickness, LV end-diastolic diameter (LVEDD) and interventricular septum diastolic thickness were determined at end-diastole. Left atrial (LA) volume and LA volume index (LAVI) were measured by biplane area-length method. Left ventricular mass was calculated by use of the Devereux formula [24]. Left ventricular hypertrophy, and pulmonary hypertension were diagnosed according to the current recommendation as stated in previous studies [23, 25]. LVEF was assessed by the biplane modified Simpson’s method [26] and values less than 50% were considered as LVSD according to a previous study [7]. LV diastolic function parameters were also collected, including mitral valve E/A ratio, annular e’ velocity, average mitral E/e’ ratio, LAVI and peak tricuspid regurgitation (TR) velocity. The investigation of body composition, for example multi-frequencies impedance meter, was not measured in this study. The laboratory parameters within 30 days of the TTE study were collected. These are; neutrophil, lymphocyte, platelet counts, hemoglobin (Hb) levels, serum albumin, calcium, phosphate and estimated glomerular filtration rate (eGFR). The eGFR was analyzed using the simplified Modification of Diet in Renal Disease (MDRD) study equation [27] or the Chronic Kidney Disease Epidemiology Collaboration (CKD-EPI) equation [28]. We measured NLR by dividing absolute neutrophil count with absolute lymphocyte count while the PLR was calculated by dividing absolute platelet with lymphocyte count. Based on previous studies [20, 29], we used limits of 3.6 and 150 of NLR and PLR for categorizing into two groups, ≤3.6 vs > 3.6 and ≤ 150 vs > 150, respectively. The corrected QT interval was also calculated with 12-lead electrocardiography using Bazett’s Formula. Peritoneal membrane function was assessed using a standard peritoneal equilibration test [30] and the dialysis adequacy was shown as total weekly Kt/V of urea.

### Statistical analysis

Intra- and inter-observer reliability of TTE were analyzed prior to the initiation of this study. The continuous data was expressed as mean with standard deviation (SD) if normally distributed, or median with interquartile range if not. Subjects were divided into 2 groups of LVSD according to the cut off value of LVEF (< 50% vs. ≥ 50%). The categorical variables were presented as frequencies and percentages analyzed using Chi-square test or Fisher’s exact test. Comparisons between groups of patients on continuous variables were performed using the Student’s t-test or Mann-Whitney test. Pearson’s and Spearman’s rank correlation were applied to investigate the trend and strength of associations between LVSD and various risk factors. The univariate and multivariate logistic regression analyses were performed to determine significant factors associated with the categorical outcome variable of LVSD.

Data analysis was initiated with a univariate logistic regression analysis in order to screen for potential candidate variables and then added only these candidates; including NLR and PLR into the same model for multivariate analysis. Because of small sample size when compared with the number of variables needed, forward selection was used for considering which variables should or should not remain in the model. The backward elimination was not performed since it may force the model to over fit as for including all variables initially. Then the model selection was done by using “sw” command in STATA and keeping NLR and PLR as study factors in the model. Beta coefficient along with odds ratio and 95% confidence interval (CI) were calculated and the *P* value < 0.05 was considered significant. All statistical analysis was done using STATA 14.2 (StataCorp LP, College Station, TX, USA) software.

## Results

Baseline clinical characteristics, laboratory and echocardiographic parameters are summarized in Table 1. Among 103 subjects, 50 (48.5%) were male and the mean age was 59.3 ± 12.7 years. The participants were stratified into 2 groups according to their LVEF; i.e., group 1 with LVEF ≥50% (*n* = 85, 82.5%) and group 2 with LVEF < 50% or LVSD (*n* = 18, 17.5%). The prevalence of LVSD was found 17.5% (95% CI, 11.21–26.21%). Preexisting CAD, DM and current smoking were 20 (19.4%), 63 (61.2%) and 23 (22.3%) patients, respectively. The mean BMI was 24.2 ± 3.6 kg/m^2^ and the median time of dialysis vintage was 12 (3, 24) months. Prior CAD (*p* = 0.02), DM (*p* = 0.03), smoking (*p* = 0.01) were significantly high while Hb levels were low in the PD patients with LVSD (Additional file 1: Table S1, Table S2)*.* The median (IQR 25, 75) of PD adequacy was assessed by the total weekly Kt/V of urea was 1.7 (1.4, 2.1). Dialysis efficiency data evaluated by total weekly Kt/V of urea was not analyzed with other factors in the final because 50% of this data was missing.

**Table 1:** Baseline demographic, laboratory and echocardiographic characteristics

Demographic and clinical	Total	NLR > 3.6	NLR ≤ 3.6	PLR > 150	PLR ≤ 150
characteristics	*n* = 103	*n* = 41	*n* = 62	*n* = 31	*n* = 72
Age, y	59.3 ± 12.7	59.61 ± 13.06	59.13 ± 12.58	57.65 ± 12.52	60.04 ± 12.82
Male, n (%)	50 (48.5)	21 (51.22)	29 (46.77)	20 (64.52)	30 (41.67)
Diabetes Mellitus, n (%)	63 (61.2)	28 (68.29)	35 (56.45)	23 (74.19)	40 (55.56)
Hypertension, n (%)	101 (98.1)	40 (97.56)	61 (98.39)	30 (96.77)	71 (98.61)
Dyslipidemia, n (%)	78 (75.7)	30 (73.17)	48 (77.42)	19 (61.29)	59 (81.94)
Smoker, n (%)	23 (22.3)	4 (9.76)	19 (30.65)	8 (25.81)	15 (20.83)
Previous CAD, n (%)	20 (19.4)	11 (26.83)	9 (14.52)	5 (16.13)	15 (20.83)
Pulmonary disease, n (%)	2 (1.9)	0	2 (3.23)	1 (3.23)	1 (1.39)
SBP, mmHg	150.7 ± 23.0	148.54 ± 21.27	152.19 ± 24.15	144.03 ± 18.77	153.63 ± 24.16
DBP, mmHg	82.6 ± 12.7	83.54 ± 15.49	82.03 ± 10.62	81.55 ± 13.55	83.09 ± 12.43
BMI, Kg/m^2^	24.2 ± 3.6	23.57 ± 3.65	24.59 ± 3.62	23.77 ± 3.18	24.36 ± 3.84
BSA, m^2^	1.20 ± 0.10	1.19 ± 0.09	1.18 ± 0.09	1.22 ± 0.09	1.18 ± 0.09
Duration of CAPD, months^a^	12.0 (3.0, 24.0)	11.0 (4.0, 23.0)	12.0 (3.0, 24.0)	9.0 (2.0, 18.0)	13.5 (4.25, 28.50)
Blood biochemistry					
NLR ^a^	3.3 (2.3, 4.3)	4.9 (3.9, 5.8)	2.5 (2.0, 3.2)	5.2 (3.4, 8.8)	2.9 (2.2, 3.6)
PLR ^a^	164.1 (119.0, 231.6)	168.1 (109.8, 246.9)	97.1 (68.9, 128.5)	203.5 (168.75, 271.8)	95.9 (68.8, 120.7)
Hb; g/dl	10.4 ± 1.8	10.4 ± 1.8	10.4 ± 1.8	10.2 ± 2.1	10.6 ± 1.6
Serum creatinine; g/dL	8.9 ± 3.1	8.7 ± 2.8	9.1 ± 3.3	8.9 ± 2.9	8.9 ± 3.2
GFR (CKD-EPI)^a^;ml/min/1.73m^2^	5.3 (4.0, 6.9)	6.1 ± 2.9	6.1 ± 5.0	6.1 ± 2.7	6.1 ± 4.8
Serum albumin; g/dL	3.4 ± 0.5	3.2 ± 0.5	3.6 ± 0.5	3.1 ± 0.6	3.6 ± 0.5
Serum calcium; mg/dL	8.4 ± 0.9	8.2 ± 1.1	8.6 ± 0.8	8.0 ± 0.9	8.6 ± 0.9
Serum phosphate; mg/dL	4.8 ± 1.5	4.6 ± 1.4	4.9 ± 1.5	4.9 ± 1.6	4.7 ± 1.4
QTc interval; msec	459.5 ± 43.2	460.4 ± 48.6	458.9 ± 39.5	468.6 ± 47.8	455.5 ± 40.7
Echocardiographic parameters					
LVSD	18 (17.48)	11 (26.83)	7 (11.29)	9 (29.03)	9 (12.50)
LVEF ^b^; %	59.5 + 10.9	56.0 ± 12.3	61.7 ± 9.4	55.2 ± 11.2	61.3 ± 10.4
LVEDD; mm	49.5 ± 8.3	49.6 ± 8.6	49.5 ± 8.2	50.9 ± 8,1	48.9 ± 8.4
LVESD; mm	32.9 ± 8.5	34.5 ± 9.3	31.9 ± 7.8	34.9 ± 8.9	32.1 ± 8.2
LAVI; ml/m^2^	51.2 ± 20.9	53.7 ± 28.7	51.3 ± 19.4	53.4 ± 22.1	51.8 ± 24.1
LVMI; g/m^2^	205.1 ± 78.7	206.9 ± 79.2	203.9 ± 78.9	216.8 ± 82.3	200.1 ± 77.2
IVSD; mm	12.1 ± 2.3	12.2 ± 2.3	11.9 ± 2.4	12.4 ± 2.7	11.9 ± 2.2
IVSS; mm	15.1 ± 2.4	15.1 ± 2.5	15.1 ± 2.4	15.5 ± 2.9	14.9 ± 2.2
Mitral E/A	0.7 (06, 0.9)	0.7 (0.6, 0.9)	0.7 (0.6, 1.1)	0.8 (0.6, 1.2)	0.7 (0.6, 0.9)
Mitral E/e’	18.5 ± 8.5	20.4 ± 10.9	17.2 ± 6.2	19.4 ± 10.4	18.1 ± 10.5
RVSP; mmHg	42.9 ± 14.1	43.6 ± 15.6	42.5 ± 13.2	45.4 ± 14.7	41.8 ± 13.8
TAPSE; cm	2.3 ± 0.5	2.1 ± 0.5	2.4 ± 0.5	2.2 ± 0.6	2.4 ± 0.5

Values are means ± SD, ^a^ values present as medians (IQR 25,75)

_*CAD* coronary artery disease, *dx* disease, *SBP* systolic blood pressure, *DBP* diastolic blood pressure, *BMI* body mass index, *BSA* body surface area, *CAPD* continuous ambulatory peritoneal dialysis, *NLR* neutrophil lymphocyte ratio, *PLR* platelet lymphocyte ratio, *Hb* hemoglobin, *GFR* glomerular filtration rate, *QTc* Corrected QT interval=_ $QT\sqrt{RR}$_, *LVSD* left ventricular systolic dysfunction, *LVEF* left ventricular ejection fraction,_^b^_LVEF by modified Simpson’s method, *LVEDD* left ventricular end diastolic diameter, *LVESD* left ventricular end systolic diameter, *LAVI* left atrial volume index, *LVMI* left ventricular mass index, *IVSD* interventricular septum in diastole, *IVSS* interventricular septum in systole, *Mitral E/A* mitral valve E velocity divided by A velocity, *Mitral E/e*’ mitral valve E velocity divided by mitral annular e’ velocity, *RVSP* right ventricular systolic pressure, *TAPSE* tricuspid annular plane systolic excursion_

The mean (SD) LVEF was 59.5% (10.9) [63.2% (7.4) versus 41.7% (6.80) in group 1 and group 2, respectively]. The left ventricular mass index (LVMI) (*p* < 0.01), LAVI (*p* = 0.03), and right ventricular systolic pressure (RVSP) (*p* = 0.01) were significantly higher in group 2. The LVEF ≤40% was observed in only 7 of them (6.8%) and that ranged from 21 to 40% (data not shown). LV dilatation was found in 12(14.1%) and 13(72.2%) patients of group 1 and 2, respectively. All patients of group 2 and 52 (61.2%) patients of group 1 have met the criteria of elevated LV filling pressure and at least grade II of LV diastolic dysfunction [31]: average mitral E/e’ ratio > 14, LAVI > 34 ml/m^2^ and peak TR velocity > 2.8 m/sec.

**Fig. 1:**
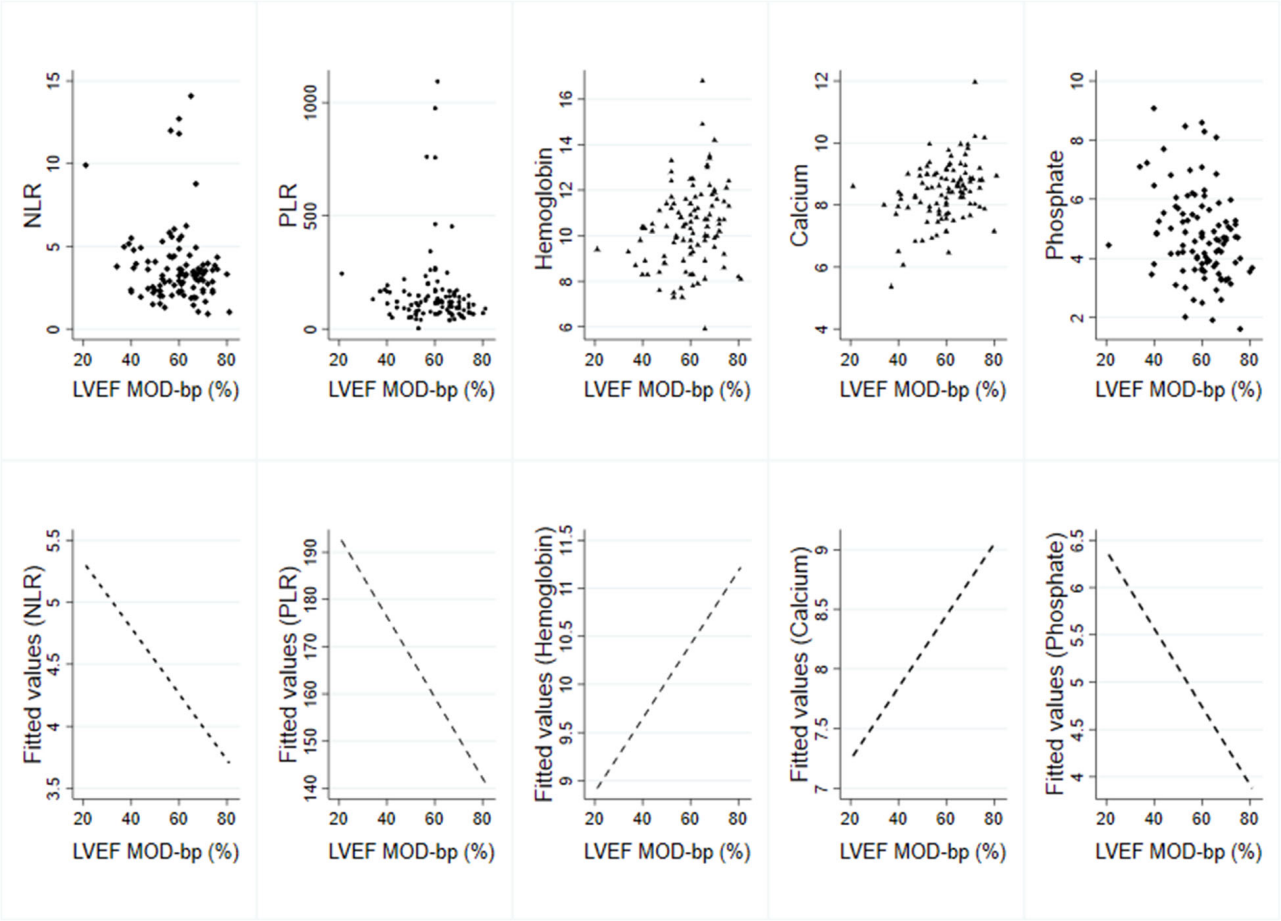
Correlation between laboratory factors and left ventricular ejection fraction (LVEF), modified Simpson's method

**Fig. 2:**
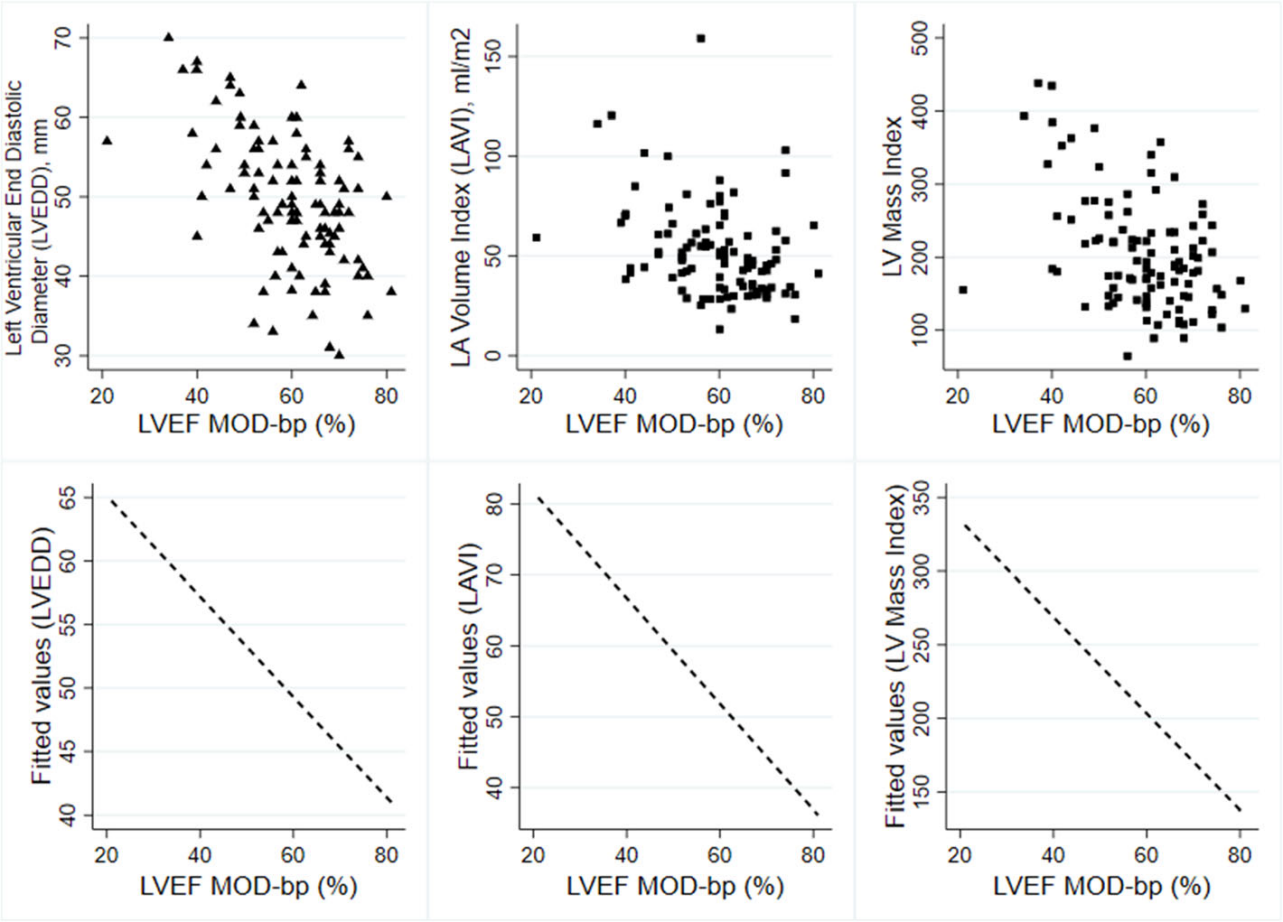
Correlation between echocardiographic parameters and left ventricular ejection fraction (LVEF), modified Simpson's method

Duration of dialysis (r = 0.22, *p* = 0.03), Hb level (r = 0.24, *p* = 0.02) and serum calcium (r = 0.35, *p* < 0.01) showed a considerably positive correlation with LVEF. The serum phosphate (r = − 0.31, p < 0.01), LVEDD (r = − 0.52, p < 0.01), LAVI (r = − 0.35, p < 0.01), LVMI (r = − 0.46, p < 0.01) and RVSP (r = − 0.29, p < 0.01) were significantly negative correlations (Table 2, Figs. 1 and 2).

**Table 2:** Correlation between LVEF and clinical and laboratory parameters

	r	*P*-value
Age, y	+ 0.169	0.088
SBP	+ 0.033	0.743
DBP	- 0.066	0.509
BMI, Kg/m^2^	- 0.024	0.813
BSA, m^2^	- 0.027	0.784
Duration of CAPD, months	+ 0.220	0.026
NLR	- 0.125	0.209
PLR	- 0.165	0.096
Hb, g/dl	+ 0.236	0.017
Serum creatinine, g/dL	- 0.185	0.062
GFR (MDRD); ml/min/1.73m^2^	+ 0.147	0.139
GFR (CKD-EPI); ml/min/1.73m^2^	+ 0.128	0.196
Serum albumin; g/dL	+ 0.188	0.058
Serum calcium; mg/dL	+ 0.347	0.003
Serum phosphate; mg/dL	- 0.305	0.002
QTc interval; msec	- 0.128	0.197
LVEDD; mm	- 0.521	< 0.001
LAVI; ml/m^2^	- 0.348	< 0.001
LVMI; g/m^2^	- 0.456	< 0.001
RVSP	- 0.294	0.003

_*LVEF* left ventricular ejection fraction, *SBP* systolic blood pressure, *DBP* diastolic blood pressure, *BMI* body mass index, *BSA* body surface area, *CAPD* continuous ambulatory peritoneal dialysis, *NLR* neutrophil lymphocyte ratio, *PLR* platelet lymphocyte ratio, *Hb* hemoglobin, *GFR* glomerular filtration rate, *QTc* Corrected QT interval=_ $QT\sqrt{RR}$_, *LVEDD* left ventricular end diastolic diameter, *LAVI* left atrial volume index, *LVMI* left ventricular mass index, *RVSP* right ventricular systolic pressure_

The relationship between LVSD and associated factors were determined by univariate and multivariate logistic regression analysis. The LVSD was found to be associated with prior CAD, DM, smoking, Hb level, NLR > 3.6, PLR > 150, high calcium and phosphate level (Table 3, all *P* < 0.05). However, in multivariate analysis showed only that the underlying CAD (OR 5.08, 95% CI: 1.16, 22.19), DM (OR 6.36, 95% CI: 1.29, 31.49), smoking (OR 10.62, 95% CI: 2.17, 51.99), NLR > 3.6 (OR 6.77, 95% CI: 1.41, 32.52), and high serum phosphate (OR 9.39, 95% CI: 2.16, 40.92) were significantly associated with LVSD (Table 3).

**Table 3:** Factors associated with LVSD (LVEF < 50%): Univariate and Multivariate analysis among PD patients

	*Univariate analysis*			*Multivariate analysis*		
Factors	Odds Ratio	95% CI	P-value	Odds Ratio	95% CI	*P*-value
Previous CAD	3.524	1.154, 10.766	0.027	5.078	1.162, 22.199	0.031
Diabetes	3.854	1.038, 14.308	0.044	6.362	1.285, 31.493	0.023
Smoke	3.733	1.263, 11.039	0.017	10.617	2.168, 51.988	0.004
NLR > 3.6	2.881	1.011, 8.207	0.048	6.774	1.411, 32.519	0.017
PLR > 150	2.864	1.008, 8.132	0.048	–	–	–
Hb ≤ 10 g/dL	3.313	1.132, 9.693	0.029	–	–	–
Calcium^a^; mg/dL	0.209	0.064, 0.691	0.010	–	–	–
Phosphate^b^; mg/dL	4.306	1.404, 13.209	0.011	9.397	2.158, 40.920	0.003

^a^ serum calcium ≥8.5 mg/dL, ^b^ serum phosphate ≥4.8 mg/dL

*LVSD* left ventricular systolic dysfunction, *LVEF* left ventricular ejection fraction, *PD* peritoneal dialysis, *DM* diabetes, *CAD* coronary artery disease, *NLR* neutrophil lymphocyte ratio, *PLR* platelet lymphocyte ratio, *Hb* hemoglobin

In addition, further analysis of PD patients with LVSD, prior CAD (OR 10.32, 95% CI: 1.71, 62.14, *p* = 0.01), and PLR > 150 (OR 10.11, 95% CI: 1.54, 66.26, *p* = 0.02), were significant variable factors associated with LVEF ≤40% (Table 4).

**Table 4:** Factors associated with LVSD (LVEF < 40%): Univariate and Multivariate analysis among PD patients

	*Univariate Analysis*			*Multivariate Analysis*		
Factors	Odds Ratio	95% CI	P-value	Odds Ratio	95% CI	*P*-value
Previous CAD	6.667	1.359, 32.701	0.019	10.316	1.712, 62.142	0.011
Diabetes	1.638	0.302, 8.877	0.567	–	–	–
Smoke	5.404	1.114, 26.205	0.036	–	–	–
NLR > 3.6	4.167	0.768, 22.606	0.098	–	–	–
PLR > 150	6.731	1.229, 36.862	0.028	10.107	1.542, 66.261	0.016
Hb ≤ 10 g/dL	3.654	0.674, 19.795	0.133	–	–	–
Calcium^a^; mg/dL	0.141	0.163, 1.216	0.075	–	–	–
Phosphate^b^; mg/dL	1.788	0.379, 8.432	0.462	–	–	–

^a^ serum calcium ≥8.5 mg/dL, ^b^ serum phosphate ≥4.8 mg/dL

*LVSD* left ventricular systolic dysfunction, *LVEF* left ventricular ejection fraction, *PD* peritoneal dialysis, *DM* diabetes, *CAD* coronary artery disease, *NLR* neutrophil lymphocyte ratio, *PLR* platelet lymphocyte ratio

## Discussion

This study provides evidence that there is association between prior CAD, diabetes, smoking, high NLR and serum phosphate levels in PD patients with LVSD. There is correlation between inflammatory markers; i.e., NLR and PLR with LVSD of PD patients. The prevalence of LVSD among PD subjects was comparable with data seen in previous studies [4, 9, 11, 14]. Prior CAD was a strong indicator for PD patients with LVEF ≤0.4 which was similar to an earlier study [14]. However, the independent factors for LVSD in this study were different which could be explained by using a distinctive definition of LVSD (0.5 vs. 0.4) and enrolled participants were not only peritoneal dialysis (25%), but also hemodialysis (75%) patients [14]. We applied LVEF < 0.5 as a cutoff value of LVSD in correspondence to a previous study [7]. This value could significantly anticipate poor survival outcomes of ESRD patients for both cardiovascular and all causes of mortality. The progressive declining of LV systolic function could be prevented from early detection of LVSD.

Neutrophils, lymphocytes and monocytes play an important role in the systemic inflammatory response to severe injury and infection. A previous study investigated an immune response to endotoxemia and has demonstrated an increased amount of circulating neutrophils and a decreased number of lymphocytes [32]. It has been documented that inflammatory cytokines lead to heart failure progression and LV remodeling by generating hypertrophy and promoting myocyte apoptosis and fibrosis [16, 33]. These can explain the predictive role of NLR for the risk of LVSD.

The serum levels of calcium and phosphate were reported to be associated with LV diastolic function in ESRD patients [34, 35]. These levels with LVSD has a similar correlation in our study. Hyperphosphatemia, observed in CKD patients, is associated with cardiac hypertrophy which may worsen cardiac contractility and heart failure [36]. Prior studies also demonstrate a relationship between serum calcium and phosphate levels with coronary, myocardial and valvular calcifications in uremic patients [37, 38]. Elevated serum phosphate levels may worsen the effects of coronary atherosclerosis through increased vascular calcification and smooth muscle proliferation [39]. This calcification may change microcirculatory hemodynamics through increased extravascular resistance, compromised myocardial perfusion and induced LV diastolic and systolic dysfunction [34].

Anemia is frequently observed in patients with CKD and could be found more than 80% of patients with a creatinine clearance < 25 ml/min/1.73m^2^ [40]. Anemia is associated complexly to the relationship between CKD and CAD in “Cardio-Renal-Anemia syndrome” [41, 42]. We postulate that there is an association as anemia causes reduced oxygen carrying capacity and contributes to myocardial ischemia and abnormal adaptive cardiac remodeling. There is significant heterogeneity in Hb level at each stage of kidney disease. Therefore, it is possible that anemia represents not only declined eGFR but also results in inflammation [43, 44]. Chronic anemia cause volume overload, which results in ventricular dilatation. Consequently, the length of the sarcomeres increases for a better overlap between myofilaments. The thickness of the left ventricle increases to counter balance the increased radius and eccentric hypertrophy which can predict poor outcomes of CAD and life expectancy [45].

The LVEF measurement was partly influenced by volume overload. Patients with LVEF < 0.5 had larger LVEDD, LVESD, LAVI and peak TR velocity, however in this study 61.2% of participants with LVEF ≥0.5 also had evidence of increased LV filling pressure, but non-significantly difference of LV dilatation.

This is the first study to address the association between these factors and LVSD in PD patients. Comparing with previous studies [10, 14], we investigated CKD patients on PD and not hemodialysis. To avoid confounding bias of LVSD outcomes, the echocardiographic factors are not included as the study factors in the final analysis. Our analysis indicated that NLR, PLR, Hb, serum calcium and phosphate levels, which are easily obtained from routine laboratory testing, may be useful for predicting and modifying the risk of LVSD in PD patients.

This study has some limitations; first, it is a cross-sectional study which could not establish the causal relationship of LVSD among those variable factors. Secondly this study was done based on a single center and limited to PD patients, thus it has a small sample size for a small event of LVSD. This may be the reason of abnormally wide CI of OR. For accepting the final model and its validity, the goodness of fit was assessed and illustrated well fit with the given data. To reduce the width of a CI, the additional time, resources and budgets are needed to collect a larger sample. Lastly, C-reactive protein (CRP), high sensitivity CRP (hs-CRP) and other inflammatory markers are not included in our analysis as a result of the measurement of CRP and hs-CRP not widely used and rather expensive. NLR and PLR could be derived from a routine simple test; i.e., complete blood count. However, previous studies have illustrated the NLR could be a potential surrogate marker of systemic inflammation in its ability to predict hs-CRP [46] and CRP [47] levels of ESRD patients. In addition, the prognostic value of CRP as indicator of LVSD from previous studies [48, 49] have shown solely in hemodialysis patients. Future studies of CRP or hs-CRP levels in PD patients should be investigated as a prognostic marker for predicting LVSD of PD patients. In addition, further longitudinal studies which include more participants and centers are needed to explore this observed relationship for both modes of dialysis, peritoneal and hemodialysis, and for long term survival.

## Conclusion

Prior history of CAD, DM, smoking, high NLR and serum phosphate levels were found to be associated with LVSD for our PD patients. The evidence from prospective study is needed to confirm the predictive value of these variables.

## Additional file

Additional file 1: **Table S1.** Baseline characteristics and Laboratory parameters in group 1 (normal LVEF) and group 2 (LVSD). **Table S2.** Echocardiographic parameters in group 1 (normal LVEF) and group 2 (LVSD). (DOCX 21 kb)

## Data Availability

All data used and/or analysed during the current study are presented in the manuscript or available from the corresponding author on reasonable request.

## Abbreviations

BMI: Body mass index
BSA: Body surface area
CAD: Coronary artery disease
CI: Confidence interval
CKD: Chronic kidney disease
CVD: Cardiovascular diseases
DM: Diabetes Mellitus
eGFR: estimated glomerular filtration rate
ESRD: End stage renal disease
Hb: Hemoglobin
HD: Hemodialysis
LA: Left atrium
LAVI: Left atrial volume index
LVEDD: Left ventricular end-diastolic diameter
LVEF: Left ventricular ejection fraction
LVESD: Left ventricular end-systolic diameter
LVMI: Left ventricular mass index
LVSD: Left ventricular systolic dysfunction
MDRD: Modification of Diet in Renal Disease
NLR: Neutrophil to lymphocyte ratio
PD: Peritoneal dialysis
PLR: Platelet to lymphocyte ratio
RVSP: Right ventricular systolic pressure
SD: Standard deviation
TR: Tricuspid regurgitation
TTE: Trans-thoracic echocardiography
