# Supplementary material for: Associations of left ventricular systolic dysfunction with the factors among Thai patients on peritoneal dialysis: a cross-sectional study
Source: BMC Nephrol. 2019 Jul 12;20:257. doi: 10.1186/s12882-019-1418-7 (PMC6625069; doi:10.1186/s12882-019-1418-7)
Supplement: Supplementary file 1 — Table S1. Baseline characteristics and Laboratory parameters in group 1 (normal LVEF) and group 2 (LVSD). Table S2. Echocardiographic parameters in group 1 (normal LVEF) and group 2 (LVSD). (DOCX 21 kb) [file 12882_2019_1418_MOESM1_ESM.docx]

**Additional File 1**

**Table S1** Baseline characteristics and Laboratory parameters in group 1 (normal LVEF) and

group 2 (LVSD)

**Table S2** Echocardiographic parameters in group 1 (normal LVEF) and group 2 (LVSD)

**Table S1**  Baseline characteristics and laboratory parameters in group 1 (normal LVEF)

and group 2 (LVSD)

|  | All PD patients  (n=103) | Group 1  (n=85) | Group 2  (n=18) | P-value |
| --- | --- | --- | --- | --- |
| Age, y | 59.3±12.7 | 60.4±12.5 | 54.4±12.9 | 0.814 |
| Male, n (%) | 50 (48.5) | 38 (44.7) | 12 (66.7) | 0.090 |
| Diabetes, n (%) | 63 (61.2) | 48 (56.5) | 15 (83.3) | 0.034 |
| Hypertension, n (%) | 101 (98.1) | 83 (97.6) | 18 (100) | 0.511 |
| Dyslipidemia, n (%) | 78 (75.7) | 64 (75.3) | 14 (77.8) | 0.823 |
| Smoker, n (%) | 23 (22.3) | 15 (17.6) | 8 (44.4) | 0.013 |
| Previous CAD, n (%) | 20 (19.4) | 13 (15.3) | 7 (38.9) | 0.022 |
| Pulmonary disease, n (%) | 2 (1.9) | 2 (2.4) | 0 (0) | 0.511 |
| SBP, mmHg | 150.7±23.0 | 151.9±23.4 | 145.4±20.6 | 0.565 |
| DBP, mmHg | 82.6±12.7 | 82.5±12.8 | 83.4±12.9 | 0.859 |
| BMI, Kg/m^2^ | 24.2±3.6 | 24.1±3.7 | 24.7±3.3 | 0.649 |
| BSA, m^2^ | 1.2±0.1 | 1.2±0.1 | 1.2±0.1 | 0.500 |
| Duration of CAPD, months* | 12.0 (3.0, 24.0) | 12.5(3.5, 28.0) | 6.75(2.0, 15.0) | 0.098 |
| LVEF^#^, % | 59.5(10.9) | 63.2(7.4) | 41.7(6.8) | 0.734 |
| NLR * | 3.3 (2.3, 4.3) | 3.2(2.3, 4.1) | 3.7 (2.4, 4.9) | 0.455 |
| PLR * | 164.1  (119.0, 231.6) | 160.2  (116.2, 210.3) | 197.1  (131.3, 275.7) | 0.106 |
| Hb, g/dl | 10.4±1.8 | 10.6±1.8 | 9.3±0.9 | 0.004 |
| Serum creatinine, g/dL | 8.9±3.1 | 8.6±2.9 | 10.4±3.3 | 0.552 |
| GFR(MDRD)*;  ml/min/1.73m^2^ | 6.0 (4.5, 7.6) | 5.6 (4.1, 6.9) | 4.9 (3.6, 6.1) | 0.238 |
| GFR(CKD-EPI)*;  ml/min/1.73m^2^ | 5.3 (4.0, 6.9) | 6.2 (4.7, 7.8) | 5.2 (4.0, 6.9) | 0.231 |
| Serum albumin; g/dL | 3.4±0.5 | 3.5±0.6 | 3.3±0.5 | 0.374 |
| Serum calcium; mg/dL | 8.4±0.9 | 8.5±0.9 | 7.9±1.0 | 0.379 |
| Serum phosphate;mg/dL | 4.8±1.5 | 4.6±1.4 | 5.6±1.6 | 0.452 |
| Corrected QT interval; msec | 459.5±43.2 | 458.4±44.4 | 464.5±37.4 | 0.426 |

_Values are means ± SD, * values present as medians (IQR 25,75), # LVEF by modified Simpson’s method_

_LVSD; left ventricular systolic dysfunction, SBP; systolic blood pressure, DBP; diastolic blood pressure, BMI; body mass index, BSA; body surface area, CAPD; continuous ambulatory peritoneal dialysis,_ _LVEF; left ventricular ejection fraction, NLR; neutrophil lymphocyte ratio, PLR; platelet lymphocyte ratio, Hb; hemoglobin, GFR; glomerular filtration rate_

**Table S2** Echocardiographic parameters in group 1 (normal LVEF) and group 2 (LVSD)

|  | All PD patients  (n=103) | Group 1  (n=85) | Group 2  (n=18) | P-value |
| --- | --- | --- | --- | --- |
| LVEF ^#^; % | 59.5 + 10.9 | 63.2 + 7.4 | 41.7 + 6.8 | 0.734 |
| LVEDD; mm | 49.5 + 8.3 | 47.5 + 7.1 | 59.1 + 6.9 | 0.999 |
| LVESD; mm | 32.9 + 8.5 | 30.4 + 6.4 | 45.1 + 6.0 | 0.809 |
| LAVI; ml/m2 | 51.2 + 20.9 | 47.2 + 17.6 | 70.0 + 25.4 | 0.029 |
| LVMI; g/m2 | 205.1 + 78.7 | 186.9 + 60.9 | 290.8 + 96.9 | 0.0054 |
| IVSD; mm | 12.1 + 2.3 | 11.9 + 2.3 | 12.8 + 2.5 | 0.532 |
| Mitral E/e’ | 18.5 + 8.5 | 17.3 + 0.8 | 24.1 + 2.9 | 0.002 |
| TR velocity; m/s | 3.2 + 0.5 | 3.2 + 0.1 | 3.6 + 0.2 | 0.001 |
| RVSP; mmHg | 42.9 + 14.1 | 40.6 + 11.9 | 53.7 + 18.5 | 0.009 |
| TAPSE; cm | 2.3 + 0.5 | 2.4 + 0.5 | 2.1 + 0.7 | 0.043 |

_Values are means ± SD, # LVEF by modified Simpson’s method_

_LVSD; left ventricular systolic dysfunction, PD; peritoneal dialysis, LVEF; left ventricular ejection fraction, LVEDD; left ventricular end diastolic diameter, LVESD; left ventricular end systolic diameter, LAVI; left atrial volume index, LVMI; left ventricular mass index, IVSD; interventricular septum in diastole, Mitral E/e’; mitral valve E velocity divided by mitral annular e’ velocity, TR; tricuspid regurgitation, RVSP; right ventricular systolic pressure, TAPSE; tricuspid annular plane systolic excursion._
